# Supplementary material for: Stimulation of THP-1 Macrophages with LPS Increased the Production of Osteopontin-Encapsulating Exosome
Source: Int J Mol Sci. 2020 Nov 11;21(22):8490. doi: 10.3390/ijms21228490 (PMC7696453; doi:10.3390/ijms21228490)
Supplement: Supplementary file 1 [file ijms-21-08490-s001.pdf]

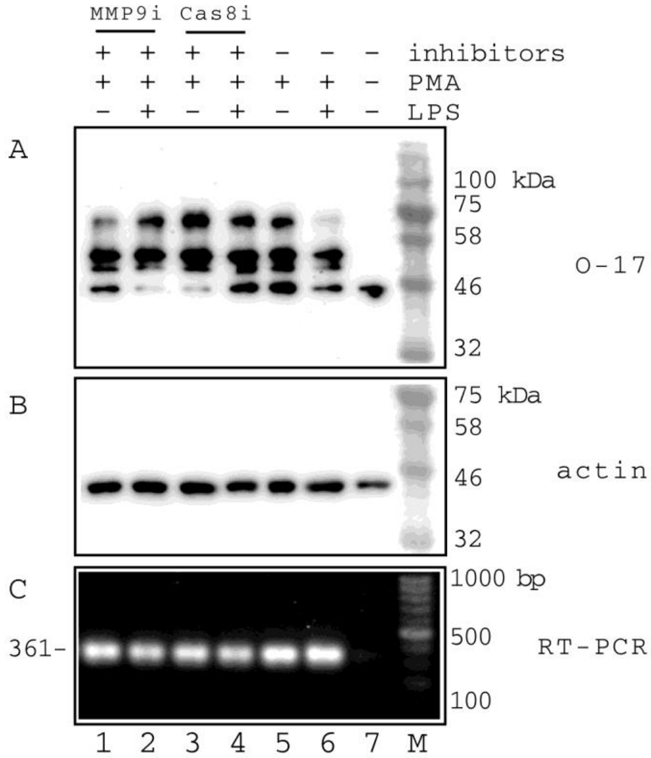

Figure S1 Effect of protease inhibitors on OPN expression and transcript in LPS-stimulated PMA differentiated THP-1 macrophages. PMA differentiated cells were simulated by LPS for 24 h. and non-stimulated cells were used as controls. Western blot (**A**,**B**) and RT-PCR (**C**) were shown. For western blot O-17 (**A**) and anti-actin (**B**) antibodies were used as described in 4.5. PMA differentiated THP-1 macrophages were cultured PMA-free medium for 24 hours followed by the addition of LPS and/or protease inhibitors for 24 h. Four micrograms of the cell lysates were subjected to SDS-PAGE (12%) followed by immunostaining. M, molecular weight marker. For RT-PCR (**C**), total RNA was extracted with SV Total isolation system. mRNA transcription of OPN variants was analysed by reverse transcription as described in 4.5. MMP9i; MMP-9 inhibitor, Cas8i; caspase 8 inhibitor, M; molecular weight marker.
